# Medical Items

**Published:** 1911-04

**Authors:** 


### MEDICAL ITEMS

### ELIXIRS DE LUXE.

Parke, Davis & Co. announce some important improvements in their line of medicinal elixirs, a line numbering more than one hundred and twenty-five preparations and highly esteemed by physicians on the score of therapeutic excellence. The improvements cited are in manufacturing processes, in the interest of palatability, permanence and physical appearance. They are set forth at some length in the current issue of Modern Pharmacy, from which these interesting extracts are taken:

"Three or four years ago, in the gradual development of our scientific staff, we secured the services of Professor Wilbur L. Scoville, a pharmacist well-known to the country and a man pre-eminent in the field of what has been termed pharmaceutical elegance. Professor Scoville may well be considered an artist in questions concerning odor, flavor and appearance of galenicals. The first task assigned to Professor Scoville was to go systematically and patiently through our entire line of elixirs—regardless of what changes were under consideration at the time. He was given carte blanche to go ahead and suggest any modifications and improvements which seemed to him necessary.

Professor Scoville at once began an exhaustive series of experiments which took him nearly three years to complete. He went over the entire line, improving here the flavor, there the color, elsewhere the odor, and in other instances the permanence of our products. How well he succeeded may be seen by comparing any one of our elixirs with others on the market. It is our honest opinion that there is no line of elixirs in the United States to-day possessing an equal degree of therapeutic efficiency which will stand up on the druggist's shelves and retain their physical properties and clearness so long as Parke, Davis & Co.'s.

"During this three years' work we have made hundred of experimental lots which have been kept under observation for a period of from six to eighteen months. The experiments have included such things as increasing and decreasing the percentage of alcohol, noting the effects of different solvents upon the stability of the elixirs, the increase and decrease in the proportion of sugar present, and the effect of acids. We have studied the effect upon permanence of the elixirs of using fluid extracts or percolating the mixed drugs direct. The matter of aging and also the use of refining agents such as egg albumen and similar proteid matters have been tested out. The essential oils and perfumes employed have been subjected to careful criticism; many of these have been changed with the idea of getting a better blend of a more agreeable flavor.

"We might sum it up by saying that we have first attempted to make our line more stable; secondly, to improve the physical properties which appeal to the eye, and thirdly, to improve the flavors which appeal to the palate. But we want it understood that in making these improvements we have not in a single instance sacrificed the medicinal activity of the preparation."

### SYPHILITIC DERELICTS.

The nervous distress of syphilitic derelicts, is not the least of their trials, and it is urgent that relief be afforded them. PASSILFORA INCARNATA (Daniel's Concentrated Tincture) is meeting with great success in subduing syphilitic nervous manifestations, in many nstances being used conjointly with the usual anti-luetic treatment. Daniel's Passiflora has such a mared influence in these casse, and is so free from the usual untoward eqects of bromides and chloral, that it is little wonder that it is forcing the latter from their former high positions in the therapeutics of nervous disorders. The reader, if a medical man, may receive a sample by addressing the laboratory of John B. Daniel, Atlanta, Ga.

### FORCED TISSUE FEEDING.

To accomplish this, the logical lan is to adopt a food product of proven merit, one that is easily digested and which adds power to the tissue. NUTROMUL (Brown's Cotton Seed Oil Emulsion) does this as nothing else, and in all conditions of tissue waste and dissipated vitality, it is the food agent above all others. It feeds strength to debilitated structures and once more charges them with normal resistance. Cotton Seed Oil contains more nourishing qualities than any other product at the profession's command. An emulsion of it, fortified by hypophosphites, as represented by NUTROMUL, is worthy every physician's attention. The Nottoc Laboratory, Atlanta, Georgia, will gladly furnish any reputable medical man with liberal samples upon request.

### TEDIOUS CONVALESCENCE.

The tediousness of conalescence of la grippe and pneumonia shows with what force the disease has attacked the vital powers. If convalescence is to be shortened and the ability of the body to resist tuberculous processes is to be added to, resort must be had to such agents as will feed the tissues and make blood. For this purpose Cord Ext. Ol. Morrhuae Comp. (Hagee) holds high favor with the profession.

A palatable preparation of cod liver oil to which are added the hypophosphites. Hagee's Cordial of the Extract of Cod Liver Oil Compound is not surpassesd as a tissue food.

### TUBERCULAR ADENTIS.

Glandular tuberculosis presents a problem to the clinician not easy of solution, for its management involves not alone the application of drugs, but also the selection of proper diet and the ordering of and obedience to a hygienic regime which may be extremely difficult of regular enforcement. Next to fresh air and sunshine, an abundance of nutritious food, cod liver oil offers the largest measure of success and is a necessary adjunct to the foregoing measures. Since the majority of these patients are children of tender years, great care in the choice of the cod liver oil product must be exercised if the physician would derive from it the fullest remedial benefits.

The essentials of a cod liver oil preparation are effectiveness and palatability ,and these qualities are surely found in Hagee's Cordial of the Extract of Cod Liver Oil Compound. For these reasons, Cord. Ext. Ol. Morrhuae Comp. (Hagee) is especially indicated in scrofulous conditions, and will prove to be the physician's most dependable selection from materia medica. It may be continued for indefinite periods.

### DOCTOR, AFTER PNEUMONIA.

After pneumonia, as a result of its destructive influence on the body, the tissues are depleted and bodily resistance is at a low point. The patient needs blood and vitality. Tuberculous processes, finding no resistance, will graft themselves on the weakened patient, who drifts from pneumonia into tuberculosis. This is the time when a reconstructive of proven power is needed, a reconstructive that will make blood and put back into the jaded tissues some of their lost vitality. NUTOMUL (Brown's Cotton Seed Oil Emulsion) will do this. It is full of nutritious elements and is vastly superior to cod liver oil as a tissue builder. The physician giving NUTROMUL to his pneumonia convalescents will be gratified at the progress they make. It is the ideal tissue food. A sample may be had by addressing the Nottoc Laboratory, Atlanta, Georgia.

---

### PASSIFLORA IN TETANUS.

In tetanus the great power possessed by antitetanic serum must not be denied the patient. As its value lies in its prophylactic power rather than its curative action, it will not be of particular service once the convulsive seizures have gripped the patient. At this period PASSIFLORA INCARNATA (Daniel's Concentrated Tincture) has been shown to have great remedial value. It calms the patient, relaxes the spasm and tends to neutralize the extreme nerve excitability. A large number of physicians have employed PASSIFLORA INCARNATA (Daniel's Concentrated Tincture) with success, and in view of the reported successes, it should be employed in every case. Give it early and in increasing dosage.

---

### PROPER MEDICATION AND CHEERFUL COMPANY.

During the past two months, we have met with more lagrippe than anything else, and the number of cases in which the pulmonary and bronchial organs have been very slightly or not at all involved, has been greater than we have noted in former invasions. On the contrary, grippal neuralgia, rheumatism and hepatitis have been of far greater frequency, while the nervous system has also been most seriously depressed.

